# Genetic and genome-wide transcriptomic analyses identify co-regulation of oxidative response and hormone transcript abundance with vitamin C content in tomato fruit

**DOI:** 10.1186/1471-2164-13-187

**Published:** 2012-05-14

**Authors:** Viviana Lima-Silva, Abel Rosado, Vitor Amorim-Silva, Antonio Muñoz-Mérida, Clara Pons, Aureliano Bombarely, Oswaldo Trelles, Rafael Fernández-Muñoz, Antonio Granell, Victoriano Valpuesta, Miguel Ángel Botella

**Affiliations:** 1 Instituto de Hortofruticultura Subtropical y Mediterránea, Universidad de Málaga-Consejo Superior de Investigaciones Científicas (IHSM-UMA-CSIC), Departamento Biología Molecular y Bioquímica, Universidad de Málaga, 29071, Málaga, Spain; 2 BioFIG - Center for Biodiversity, Functional & Integrative Genomics, Departamento de Biologia, Universidade do Minho, Campus de Gualtar, Braga, Portugal; 3 Computer Architecture Department, University of Malaga, Campus de Teatinos, 29071, Málaga, Spain; 4 Instituto de Biología Molecular y Celular de Plantas, Consejo Superior deInvestigaciones Científicas, Universidad Politécnica de Valencia, 46022, Valencia, Spain; 5 Boyce Thompson Institute for Plant Research, Tower Road, Ithaca, NY, 14853, USA; 6 Instituto de Hortofruticultura Subtropical y Mediterránea, Universidad de Málaga-Consejo Superior de Investigaciones Científicas (IHSM-UMA-CSIC), Est. Exp. La Mayora, Algarrobo-Costa, Málaga, Spain; 7 Departamento de Biología Molecular y Bioquímica, Universidad de Málaga, Málaga, Spain

## Abstract

**Background:**

L-ascorbic acid (AsA; vitamin C) is essential for all living plants where it functions as the main hydrosoluble antioxidant. It has diverse roles in the regulation of plant cell growth and expansion, photosynthesis, and hormone-regulated processes. AsA is also an essential component of the human diet, being tomato fruit one of the main sources of this vitamin. To identify genes responsible for AsA content in tomato fruit, transcriptomic studies followed by clustering analysis were applied to two groups of fruits with contrasting AsA content. These fruits were identified after AsA profiling of an F8 Recombinant Inbred Line (RIL) population generated from a cross between the domesticated species *Solanum lycopersicum* and the wild relative *Solanum pimpinellifollium*.

**Results:**

We found large variability in AsA content within the RIL population with individual RILs with up to 4-fold difference in AsA content. Transcriptomic analysis identified genes whose expression correlated either positively (*PVC* genes) or negatively (*NVC* genes) with the AsA content of the fruits. Cluster analysis using SOTA allowed the identification of subsets of co-regulated genes mainly involved in hormones signaling, such as ethylene, ABA, gibberellin and auxin, rather than any of the known AsA biosynthetic genes. Data mining of the corresponding *PVC* and *NVC* orthologs in Arabidopis databases identified flagellin and other ROS-producing processes as cues resulting in differential regulation of a high percentage of the genes from both groups of co-regulated genes; more specifically, 26.6% of the orthologous PVC genes, and 15.5% of the orthologous NVC genes were induced and repressed, respectively, under flagellin22 treatment in *Arabidopsis thaliana*.

**Conclusion:**

Results here reported indicate that the content of AsA in red tomato fruit from our selected RILs are not correlated with the expression of genes involved in its biosynthesis. On the contrary, the data presented here supports that AsA content in tomato fruit co-regulates with genes involved in hormone signaling and they are dependent on the oxidative status of the fruit.

## Background

L-ascorbic acid (AsA; vitamin C) is essential for most of the living plant tissues. In addition to its known function as an antioxidant, AsA has important roles in plant cell growth and expansion, photosynthesis, and hormonal regulation [1-3]. Humans are unable to synthesize AsA due to mutations in the enzyme L-gulono-1,4-lactone oxidase (GLDH) that catalyzes the final step of its biosynthesis, and as consequence need to incorporate AsA through the dietary consumption of fresh fruits and vegetables (4). Although AsA deficiency is not a current problem in developed countries, it is recognized that high AsA dietary consumption has important health benefits for the consumer, and an increased intake of AsA has been associated with a decreased incidence of several important human diseases and disorders [4-6].

So far, the study of genes regulating AsA content in plants has been tackled mainly through the use of reverse genetics targeting AsA biosynthesis and recycling genes [7-19]. Even though classical genetic approaches has resulted in the cloning of genes responsible for complex traits [20,21], and several QTLs have been identified for AsA, yet the genes underlying these QTLs need to be determined [22,23]. This is likely because identification of the gene responsible for a QTL is time-consuming and technically demanding especially when the trait requires careful analytical techniques as it is the case for AsA.

In order to overcome these drawbacks, the use of omics approaches to study complex traits have emerged during the past years, and the use of global transcript abundance together with gene cluster analysis has become a useful approach to predict and in some cases assign gene functions [24-27]. The premise behind clustering analysis is that genes having similar expression profiles across a set of conditions (e.g., tissue type, time-point series during development, responses to different stresses) may share similar functions or be involved in similar processes [28]. Still, it is clear that genes with the similar functions do not necessarily share similar transcriptional patterns, and conversely. Despite this, it has been shown that large numbers of functionally related genes show very similar expression patterns under a relevant set of conditions, especially genes that are co-regulated by common transcription factors, or whose products are components of multiprotein complexes [29]. For these reasons, clustering genes with similar expression patterns may allow to assign putative functions to unknown genes via “guilt-by-association” [29]. In order to accomplish this, several clustering techniques such as hierarchical clustering (HC) [27], self-organizing map (SOM) [24,25], and self-organizing tree algorithm (SOTA) [26] have been successfully used in tomato to identify genes with correlated expression that turned out in the selection of candidate genes for further functional analyses. As an example, the transcriptomic comparison of a *S. pennellii* introgression line (IL) with its recurrent *S. lycopersicum* parental line, identified a pectinesterase and two polygalacturonase genes that were associated to the different AsA accumulation in the IL fruits [27].

In this study, several genes that correlate with AsA content in tomato fruit have been identified through the use of an algorithm designed to draw functional associations among differentially expressed genes. These possible associations are discussed in terms of the physiological processes that might ultimately regulate the AsA content in tomato fruits, and by mining Arabidopsis expression data repositories we identify cues that may be triggering expression changes similar to those associated with the AsA content in red tomato fruits.

## Results and discussion

### Identification of tomato RILs with contrasting content of AsA in ripe fruits

**Figure 1:**
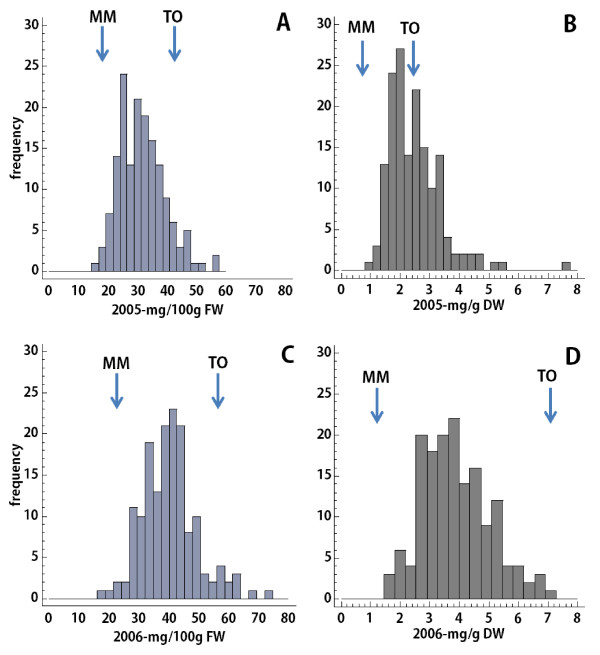
**Frequency distribution of fruit AsA content in the 158 RILs during two harvests (years 2005 and 2006).** AsA content is expressed as fresh weight (FW; mg/100 g) and dry weight (DW; mg/g). **A** and **B** show AsA content in 2005, expressed as FW and DW, respectively. **C** and **D** show AsA content in 2006, expressed as FW and DW, respectively. AsA content in parental *S. lycopersicum* cv. Moneymaker (MM) and S*. pimpinellifollium* acc. TO-937 (TO) lines is indicated with arrows in the plots.

A population of 158 *Recombinant Inbred Lines* (RILs) (F8) generated from an inter-specific cross of the domesticated species *Solanum lycopersicum* cv. Moneymaker (MM) and the wild species *Solanum pimpinellifollium* (accession TO-937) has been evaluated for AsA content in two consecutive years (Figure 1). Pericarp tissue from at least three red ripe fruits per plant and three plants per genotype were evaluated for AsA content using High Performance Liquid Chromatography (HPLC). Figure 1 shows the distribution of AsA contents in the RIL population when expressed as fresh weight (FW) and dry weight (DW) for two years harvests. As expected, a continuous AsA content distribution was observed due to the polygenic nature of AsA accumulation [22,23]. Table 1 shows the AsA content in the parental and the range in the RILs over the two years evaluated.

**Table 1:** **Average AsA content in the parents of the RILs,*S. lycopersicum*cv. Moneymaker and S*. pimpinellifollium*acc. TO-937**

**Accession**	**AsA - Year 2005**		**AsA - Year 2006**	
	**mg/100 g FW**	**mg/g DW**	**mg/100 g FW**	**mg/g DW**
Moneymaker (*S. lycopersicum*)	14.67 ± 2.83	0.67 ± 0.08	20.22 ± 1.76	1.21 ± 0.22
TO-937 (*S. pimpinellifollium*)	33.01 ± 3.33	2.34 ± 0.74	42.45 ± 8.29	7.02 ± 2.31
RILs Range	15.92 ± 3.54 - 56.81 ± 21.85	1.01 ± 0.28 - 7.56 ± 0.91	18.01 ± 8.03 - 74.01 ± 8.21	1.60 ± 0.68 - 7.16 ± 2.40

Range in the RIL population progeny expressed as Fresh (FW) and Dry weight (DW).

Similar to previous reports on another tomato wild relative *S. pennelli*[22], the AsA content of *S. pimpinellifollium* was higher than that of the cultivated tomato *S. lycopersicum* MM by about 2-fold on a fresh weight basis (FW), and increased to around 6-fold when expressed as dry weight (DW) (Table 1). The AsA content was lower in both parents in the year 2005 and the same was true for the RILs when expressed on a FW basis indicating an important environmental influence in this trait (Table 1 and Figure 1). Thus, AsA content ranged from 15.9 mg/100 g FW to 56.8 mg/100 g FW in the year 2005 and from 18.0 mg/100 g FW to 74.0 mg/100 g FW in the year 2006 (Figure 1 and Table 1). AsA content in the population remained significantly -although weakly- stable as assessed for repeatability by the correlation coefficient (ρ = 0.29; *p-value* = 0.0003). However, when results were expressed relative to DW, the range of AsA remained approximately the same but the AsA content in most of the RILs were not consistent during both years (ρ = 0.18; *p-value* = 0.021).

**Figure 2:**
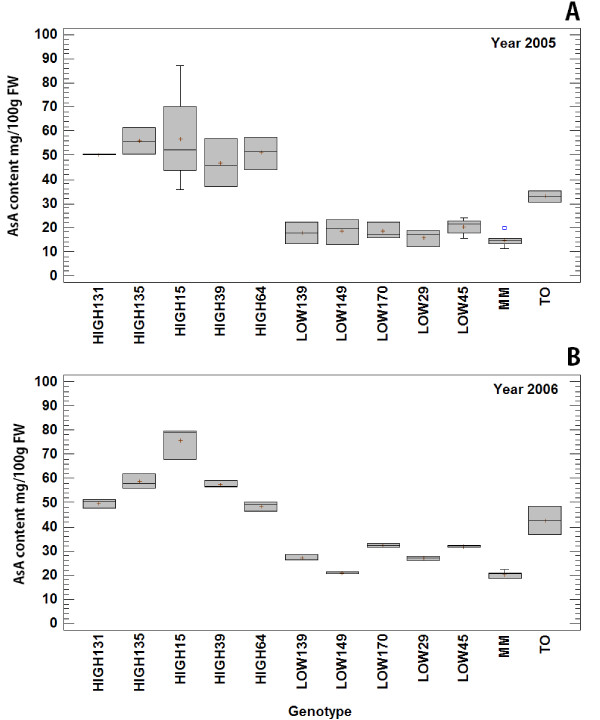
**Fruit AsA content in ten contrasting RILs selected for microarray analysis in harvest 2005 (A) and 2006 (B) and the parents*Solanum lycopersicum*cv.** Moneymaker (MM) and *Solanum pimpinellifollium* acc. TO-937 (TO). Box-and-Whisker plots were drawn extending from the *lower quartile* of the sample to the *upper quartile* covering 50% of data values. The horizontal line represents the *median* and the plus sign represent the sample *mean*. The blue square represents an outlier.

Figure 2 depicts the ten RILs lines with the most extreme values of AsA based on the AsA/FW values consistently obtained for the two years. A two-way ANOVA with data from the ten RILs selected and the parents ‘Moneymaker’ MM and TO-937, using “Genotype” and “Year” as factors, reported significant effect of Genotype (P < 0.001) and Year (P < 0.001) and no significant interaction between them (P = 0.33) (Table 2). The Genotype explained almost 83% of the variance [(Sum of Squares of Genotype/ Sum of Squares Total)*100 = (1.97E4/2.38E4)*100 = 82.77%] validating the selection of these ten RILs for further analysis (Table 2).

**Table 2:** Analysis of Variance for AsA content of ten RILs that comprise the High and Low sets of contrasting lines

***Source***	***Sum of Squares***	***DF***	***Mean Square***	F-Ratio*	***P-Value***
Main Effects					
G:Genotype	1.97 × 10^4^	11	1.79 × 10^3^	40.25	0.0000
Y:Year	971.0	1	971.0	21.86	0.0000
Interactions					
G xY	575.0	11	52.3	1.18	0.3269
Error	2.22 × 10^3^	50	44.4		
Total (corrected)	2.38 × 10^4^	73			

*All F-ratios are based on the residual mean square error. **-** Type III Sums of Squares.

### Identification of Genes whose expression correlated with AsA content in tomato fruits

Next, we aimed to identify tomato genes whose expression correlated with the AsA content in red ripe fruits from the ten RILs previously selected. For that purpose, differential transcript abundance was analyzed using two-color hybridizations and the TOM2 array (13056 spots), in which according to the unigene set #3 of the *Sol Genomic Network* (SGN; http://solgenomics.net/) 12,020 tomato sequences are represented [30].

Probes were obtained from: the five individual RILs that contained high AsA, the five individual RILs that contained low AsA, plus two probes generated with a mix of the five of each set. Three biological replicates were hybridized and normalized using as a common reference a sample containing a bulk RNA including all high and low AsA content RILs, and dye-swap was performed. Our analysis resulted in the identification of a number of genes whose expression profile correlated with the high or low AsA content in the 10 RILs (Figure 2).

Using a strict criteria in the Significant Analysis of Microarray (*SAM*; using a *d* = 1.3 and a fold-change threshold of 1, using a matrix of Z-score values) we identified 137 genes with at least 2-fold change in expression between RILs having high and low AsA content (see methods). Forty-four *PositiveVitaminC* co-regulated (*PVC*) genes (i.e. showing high expression in high-AsA RILs and low expression in low AsA RILs) and 93 *NegativeVitaminC* co-regulated (*NVC*) genes (i.e. highly expressed in low-AsA RILs and with low expression in high-AsA RILs) were identified ( Additional file 1). In order to validate the microarray results, QRT-PCRs were performed in one of each group of two contrasting RILs for several genes showing differential expression in the microarray ( Additional file 2). The QRT-PCR expression of the selected genes was in accordance with microarray data and followed the same trends in the individual lines analyzed.

### Functional classification of the *PVC* and *NVC* genes

**Figure 3:**
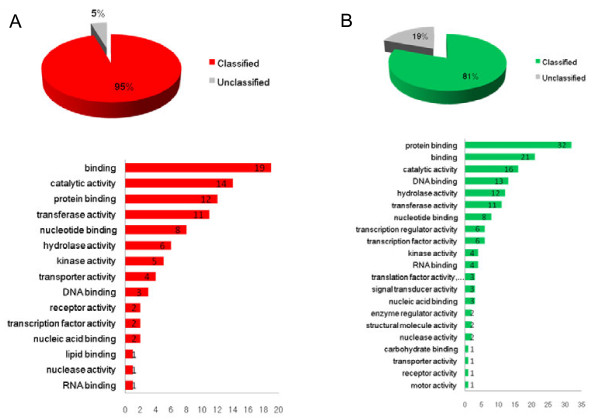
**Functional analyses of the genes upregulated in the group of high AsA containing RILs.A**) Percentage of annotated unigenes. **B**) Functional categorization of genes according to the Tomato Expression Database (TED). Numbers on bars represent the number of genes in this category.

The 137 differentially expressed genes were classified by functional categories according to the GO slim tool available in the *Tomato Expression Database* (Figure 3). The functional categorization of the 44 *PVC* genes showed that 8 genes were classified as nucleotide binding, 5 genes encoded proteins showing homology to kinases, and 4 genes encoded putative transporters. Among the 93 *NVC* genes, 6 were classified as transcription factors, 4 were classified as kinases, and one as a transporter (Figure 3).

The tomato TOM2 microarray contains oligonucleotides against 18 genes encoding for enzymes of the Wheeler-Smirnoff pathway, the main AsA biosynthetic pathway in tomato [31] and for all 5 genes involved in AsA recycling. Our results fail to identify among the differentially expressed *PVC* or *NVC* candidates any of the above-mentioned genes indicating a lack of co-regulation between the AsA metabolic pathway and the AsA content in red tomato fruits from our RIL population ( Additional file 1). This result is in accordance with a previous study showing that AsA biosynthetic genes are not the main regulators of AsA content in red tomato fruit [27,31]. However, we cannot discard that genes involved in AsA biosynthesis or recycling determine the AsA accumulation during the fruit ripening. Nevertheless we paid special attention to biosynthetic genes whose expression has been suggested to control AsA content during ripening such as *GPP1*[31], *GGP1* and *GGP2*[9,32]. However we did not find any differential expression of these genes that might account for the reported content in AsA among the RILs, indicating that their expression do not determine the final AsA content in tomato fruit in the genetic background of our RILs population. *MDHR3*, involved in AsA recycling, has been previously reported as a likely candidate for being QTL for AsA content in tomato fruit in studies with the *S. pennellii* IL population [23,33]. However, it was not identified as differentially expressed in the RIL population here analyzed. Therefore, whether the genes regulating AsA content are species-dependent or the result of polymorphisms eventually resulting in differences in enzymatic activity deserves further investigation.

Based on our results we hypothesize that the genetic factors determining the final AsA content in ripe tomato fruits in our RIL population could be the result of altered expression of genes other than AsA biosynthesis, and the analysis of the functional categories classification from the *PVC* and *NVC* genes should help us to identify those genes.

### Clustering of co-regulated genes using a Self-Organizing Tree Algorithm SOTA

**Figure 4:**
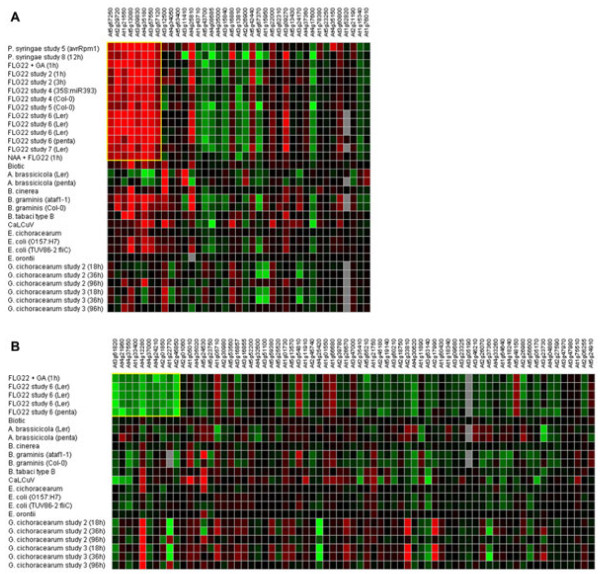
**Genes differentially expressed by microarray analyses and expression profile of candidate genes selected from the different clusters.A**) Heat map shows genes upregulated in RILs having the highest AsA content and expression profile of some candidate genes selected by SOTA clustering of these genes. **B**) Heat map shows genes downregulated in RILs having the lowest AsA content and expression profile of some candidate genes selected by SOTA clustering of these genes [70-72]. Candidate genes were selected based on their expression profile and biological relevance from the eleven clusters.

In order to identify relationships among differentially expressed genes and the AsA content in tomato we performed a Self-Organizing Tree Algorithm (SOTA) [34] using Pearson as metrics. These analyses grouped the transcript abundance profiles in 11 clusters: 4 clusters grouped the 44 *PVC* genes (Figure 4A) and 7 clusters grouped the 93 *NVC* genes (6 clusters shown in Figure 4B). The identity of the genes belonging to each cluster is shown in Table 3, which also includes the Unigene annotation based on the Sol Genomic Networks web site (http://solgenomics.net), and the corresponding Arabidopsis ortholog identified using Blast2go [35]. Next, we analyzed in more detail the identity and relationships among members belonging to individual *PVC* and *NVC* clusters. 

**Table 3:** Differentially expressed grouped by clusters obtained through SOTA analysis

**CLUSTER**	**SGN _ID #3**	**SGN _ID #2**	**ANNOTATION**	**TAIR9_BEST_MATCH**	**SGN_LOCI**
CLUSTER1	U213790	U581507	acidic extracellular 26 kD chitinase	AT3G12500.1 (1e-74)	Z15141
CLUSTER1	U219231	U584749	adenylate kinase 1, putative	AT5G63400.1 (8e-113)	None.
CLUSTER1	U220589	U585017	ABI2, protein phosphatase 2c, putative	AT3G11410.1 (1e-27)	None.
CLUSTER1	U221533	U585018	ABI2, protein phosphatase 2c, putative	AT3G11410.1 (3e-66)	None.
CLUSTER2	U213043	U579445	Solanum lycopersicum xyloglucan endotransglucosylase-hydrolase XTH3	AT4G25810.1 (6e-113)	xth3
CLUSTER2	U214169	U574723	putative auxin-regulated protein	AT2G28150.1 (6e-19)	None.
CLUSTER2	U219999	U577993	Solanum lycopersicum auxin-regulated IAA3 (IAA3)	AT5G43700.1 (3e-57)	iaa3
CLUSTER3	U214077	U581654	ascorbate peroxidase, putative	AT4G35000.1 (6e-126)	None.
CLUSTER3	U216895	U576606	ATP-binding protein serine-threonine kinase, putative	AT5G42440.1 (4e-96)	None.
CLUSTER3	U219786	U580500	WRKY-type DNA binding protein, putative	AT5G13080.1 (2e-45)	None.
CLUSTER3	U221153	U571305	protein kinase, putative	AT3G09830.2 (5e-54)	None.
CLUSTER4	U212755	U581433	glutathione-S-transferase, putative	AT3G09270.1 (3e-52)	None.
CLUSTER4	U212761	U581313	rieske iron-sulfur protein-like, putative	AT5G13430.1 (9e-109)	None.
CLUSTER4	U213289	U568366	guanylate kinase 1, putative	AT3G57550.2 (9e-118)	None.
CLUSTER4	U213865	U577749	glutathione reductase, putative	AT3G24170.1 (0)	None.
CLUSTER4	U214481	U579446	Glutathione-S-transferase, putative	AT1G78380.1 (8e-86)	None.
CLUSTER4	U218918	U577701	Gibberellin 3-beta-dioxygenase 2–3, putative	AT1G52820.1 (9e-19)	None.
CLUSTER4	U230452	U575457	Receptor-like protein kinase 5, putative	AT3G57120.1 (2e-44)	None.
CLUSTER5	U214682	U576104	MYB-CC type transcription factor, putative	AT2G01060.1 (4e-75)	None.
CLUSTER5	U214919	U577773	Solanum lycopersicum 1-aminocyclopropane-1-carboxylate oxidase	AT1G05010.1 (3e-132)	EF501822
CLUSTER5	U215104	U578524	Rac-like GTP-binding protein, putative	AT4G35020.1 (7e-95)	None.
CLUSTER5	U215556	U575819	Solanum lycopersicum ER33 protein	AT1G05710.3 (2e-39)	er33
CLUSTER5	U215557	U575819	Solanum lycopersicum ER33 protein	AT1G05710.3 (2e-39)	er33
CLUSTER5	U216238	U586247	ASKdZeta (Arabidopsis SHAGGY-related protein kinase dZeta), putative	AT2G30980.1 (0)	SlSK
CLUSTER6	U219631	U581955	Solanum lycopersicum gibberellin 20-oxidase-3	AT4G25420.1 (8e-138)	AF049900
CLUSTER6	U230811	U563226	tetratricopeptide repeat-containing protein, putative	AT1G33400.1 (3e-59)	None.
CLUSTER7	U220976	U572513	F-box protein GID2, putative	AT4G24210.1 (2e-21)	None.
CLUSTER7	U222417	U566338	bZip family Transcription factor , putative	AT5G65210.2 (3e-116)	None.
CLUSTER8	U213679	U575872	Solanum lycopersicum ETAG-A3	AT2G01850.1 (2e-131)	ETAG-A3
CLUSTER8	U218217	U570030	Solanum lycopersicum squamosa promoter binding-like protein	AT2G33810.1 (3e-33)	None.
CLUSTER8	U232883	U594117	gigantea, putative	AT1G22770.1 (4e-28)	None.
CLUSTER9	U217197	U586453	Solanum lycopersicum green ripe-like 1	AT2G26070.1 (8e-87)	grrl1
CLUSTER9	U220900	U566888	UDP-sugar transporter, putative	AT4G32272.1 (5e-136)	None.
CLUSTER10	U213278	U580739	CBL-interacting serine/threonine-protein kinase (cipk3), putative	AT2G26980.3 (0)	None.
CLUSTER10	U214406	U585891	cellulose synthase, putative	AT5G05170.1 (0)	None.
CLUSTER10	U215382	U580011	BR1, XET1 (xyloglucan endo-transglycosylase precursor)	AT3G23730.1 (2e-121)	br1
CLUSTER10	U228865	U564048	cytochrome P450, putative	AT5G24910.1 (1e-55)	None.

Unigene annotations based on the Sol Genomic Networks web site (http://solgenomics.net), and the accession numbers corresponding to Arabidopsis orthologs identified using Blast2go [35] are shown for those genes whose expression is depicted in Figure 4.

**Figure 5:**
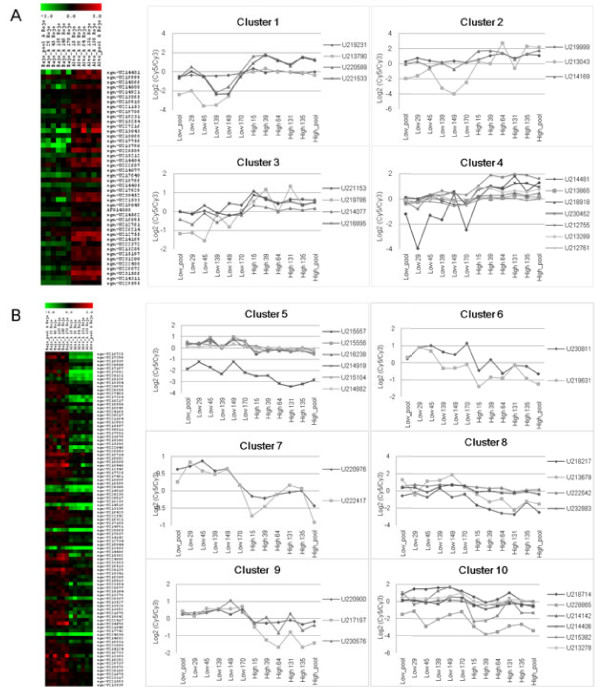
**Functional analyses of the genes downregulated in the group of high AsA containing RILs.A**) Percentage of annotated unigenes. **B**) Functional categorization of genes according to the Tomato Expression Database (TED). Numbers on bars represent the number of genes in this category.

Among the genes found in Cluster 1 there were two with homology to *ABI* genes (U220589 and U221533), a guanylate kinase, and an ethylene responsive *chitinase B*[36] (Figure 5A, Table 3). *ABI* genes encode protein phosphatases 2 C (PP2C) that are negative regulators of ABA responses through their binding and dephosphorylation of SnRK2, a core component of the cytoplasmic ABA receptors [37,38]. The connection between ABA and AsA has been previously established because concomitant upregulation of ABI2 and enzymes from the ascorbate-glutathione cycle has been recently reported in ABA-treated *A. thaliana*[39]. 

Cluster 2 contains two auxin-regulated proteins, one of them, the Aux/IAA transcription factor *Sl*AA3 (SGN-U219999) shows an ethylene ripening-associated expression pattern [40], and its transcript accumulation is dramatically reduced in the tomato ripening mutants *rinnor*, and *Nr*[41]. The *rinnor*, and *Nr* mutants lack the capacity to respond to the autocatalytic production of ethylene and to further undergo normal ethylene-regulated ripening processes [41]. Therefore, it has been proposed that, ethylene modulates auxin responsiveness during tomato fruit ripening through *Sl*AA3 [40]. The second auxin regulated protein is the *Xyloglucan endotransglucosylase-hydrolase-3* (*XET3*) gene which is expressed during fruit ripening and its expression peaks at the red stage concomitantly with the maximum of ethylene production [42]. The second auxin regulated protein is the *Xyloglucan endotransglucosylase-hydrolase-3* (*XET3*) gene which is expressed during fruit ripening and its expression peaks at the red stage concomitantly with the maximum of ethylene production [42].

Clusters 3 and 4 were closely related and grouped elements that likely have a direct role in the accumulation of AsA such as an ascorbate peroxidase, a putative chloroplastic glutathione reductase, two glutathione-S-transferases (GST), and several kinases. The up-regulation of a putative glutathione reductase together with the consumption of AsA (given the up-regulation of detoxifying enzymes requiring AsA such as GSTs) suggests that the high AsA RILs could also have higher AsA recycling rates. These results contrast with a previous study reporting that a putative ascorbate peroxidase and a putative glutathione-S-transferase genes were down-regulated in an inbred line selected for its high AsA content when compared with the parental line [27]. The different results derived from both studies suggest that AsA recycling rate and AsA content might be uncoupled events in the tomato RILs here employed.

The 93 genes down-regulated in the RILs group with the highest content of AsA could be grouped in 7 clusters (Figure 5B, Table 3 and Additional file 1). Cluster 5 includes the *S. lycopersicum 1-aminocyclopropane-1-carboxylate* (*ACC*) *oxidase* gene (SGN-U214919), whose product is an enzyme involved in ethylene biosynthesis that uses AsA as a cofactor. The silencing of the *ACC oxidase* gene in tomato is responsible for up to 87% reductions of ethylene in ripening fruits [43,44]. The ACC oxidase down-regulation together with the concomitant down-regulation of a bHLH transcription factor Ethylene Responsive 33 (ER33, SGN-U215556 and SGN-U215557) found in the same cluster seems to point to a reduced ethylene production in the RILs with higher AsA content. This is further supported by the down-regulation of a gene coding for a *S. lycopersicum* squamosa promoter binding-like (*SPBL*, SGN-U218217) protein in Cluster 8, and the *S. lycopersicum* green ripe-like 1 (*GRL1*; SGN-U217197) in Cluster 9. *SPBL* is the gene affected in the tomato Colorless nonripening (*Cnr*) mutant that results in colorless fruits with a substantial loss of cell-to-cell adhesion [45]. *GRL1* encodes for the closest homolog in tomato of the *Green Ripe* gene responsible for the non-ripening phenotype found in the dominant *Green-ripe* (*Gr*) tomato mutant [46]. Both, *SPBL* and *GRL1* are thought to be involved in ethylene responses during tomato fruit ripening, being major regulatory elements in the network controlling the process. In order to discard possible differences in the ripening stage between the two groups of contrasting AsA genes that could account for the differential expression of ethylene-related genes we searched for their expression pattern during ripening in the Tomato Expression Database (TED, http://ted.bti.cornell.edu/) or in the literature. For instance, according to TED, the expression of *S. lycopersicum 1-aminocyclopropane-1-carboxylate* (*ACC*) *oxidase* gene (SGN-U214919) does not vary significantly between breaker and red stage. On the other hand, it has been reported that the expression of ER33 (SGN-U215556 and SGN-U215557) is induced in red stage and does not vary from mature green to turning [47], so if the differential expression were due to differences in the ripening stage, they should have occurred in all RILs from one group, something highly unlikely. These data allowed us to discard differences in ripening stages as the source of differential expression in ethylene-related genes.

Clusters 6 and 7 include different elements involved in the responses to gibberellins, such as the *S. lycopersicum gibberellin 20-oxidase-3* gene and the putative *GID2* F-box gene. The Gibberellin 20-oxidase-3 is one of the three GA 20-oxidases (GA 20-ox) involved in the 13-hydroxilation gibberellin biosynthesis pathway. In this pathway, the intermediate GA_12_ is converted to GA_20_, to be subsequently hydroxilated to the bioactive GA_1_ form by GA 3-ox [48]. GA 20-oxidases are main regulatory control points of the gibberellin biosynthesis pathway [49]. The rice GID2 F-box protein is part of the SCF (SKP1, CDC53, F-box protein)-type E3 ubiquitin ligase SCF^GID2/SLY1^ that positively regulates gibberellin signaling through the degradation of specific DELLA repressors [50,51]. The differential regulation of genes implicated in gibberellin biosynthesis, with positive correlation of GA-3 ox in Cluster 4 and negative correlation of GA-20 ox in Cluster 6 is remarkable, opening the possibility that GA influences, points to a non-previously described interaction between gibberellin and AsA content in tomato.

Cluster 9 contains a gene encoding for a putative UDP-sugar transporter (SGN-U220900) (Figure 4B and Table 3). How AsA transport occurs between compartments of the cell and the apoplast remains largely unknown, as well as the transport of the several intermediates for its synthesis [52]. In many cases, NDP-sugars involved in AsA biosynthesis, such as UDP-glucose, UDP-glucuronic acid and GDP-mannose, are available in the cytosol, and likely require specific transporters to pass through the endomembrane system for further processing. The dow-nregulation of an UDP-sugar transporter in the RILs containing more AsA might be indicative of the importance of NDP-sugar transporters in the final content of AsA in a plant tissue.

**Figure 6:**
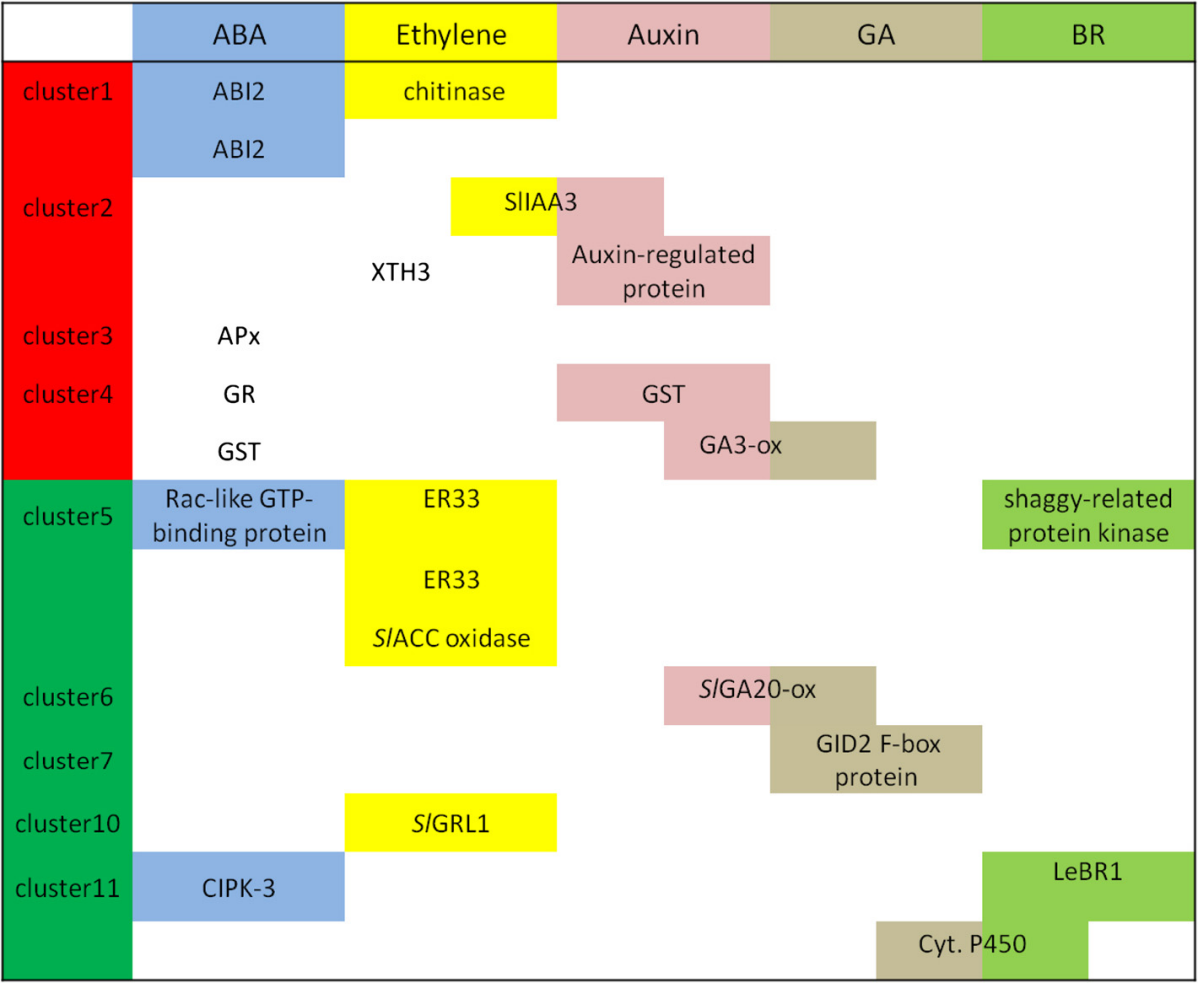
**Diagram of hormone-related candidate genes.** The candidate genes known to be related to hormone pathways are depicted with a color code and according to their cluster. Genes probably related to a hormone pathway are depicted under the hormone column but without background color. Genes in boxes sharing two colors correspond to those located at interlinking hormone pathways. Clusters with red background are those grouping genes upregulated in RILs showing the high AsA content and those with green background correspond to those grouping downregulated genes in the same RILs. Gene names are in Table 3. APx, Ascorbate Peroxidase; CIPK-3: calcineurin B-like-interacting protein kinase 3; Cyt. P450: cytochrome P450; ER33: Ethylene responsive 33; GA3-ox: Gibberellin 3 oxidase; SlGA20-ox:; S. *lycopersicum* Gibberellin 20 oxidase; GST: Glutathione-S-transferase; GR, Glutathione Reductase; LeBR1: *Lycopersicum esculentum* brasinoesteroid-regulated xyloglucan endo-transglycosylase 1; SlACC: *S. lycopersicum* 1-aminocyclopropane-1-carboxylate; SlGRL1: Green ripe-like 1; SlIAA3: Aux/IAA transcription factor 3; XTH3: Xyloglucan endotransglucosylase-hydrolase-3.

Cluster 10 groups two cell wall related genes, a putative cellulose synthase and a xyloglucan endotransglycosylase precursor *SlXET1* induced by brassinoesteroids [53]. Other cell wall modifying enzyme, the *S. lycopersicum* endoxyloglucan transferase (*EXGT-A3*), was identified in Cluster 8. This result might reveal a link between cell wall modification, degradation, and AsA content [3]. Normally, *SlXET1* appears strongly down-regulated at the beginning of turning stage of fruit ripening and appears undetectable at the red stage [42], in contrast to *SlXET3* (up-regulated in this study) whose expression drops at the turning and pink stages, to raise again at the red stage. Finally, a putative cytochrome P450 whose expression pattern resembles that of *SlXET1* was also identified in Cluster 10. Cytochromes P450, many of which are involved in the biosynthesis and metabolism of brassinosteroids [54], are known to control the redox status of the cell [55]. The fact that several elements controlling the plant redox status were identified in our analysis lead us to hypothesize that this process might be determinant for the AsA accumulation in tomato fruit (Figure 6). 

### Genes correlated with AsA content in tomato fruit are regulated by oxidative stress

Databases for co-expression analysis have been established for the model plant *Arabidopsis thaliana*, which can be used to get insights into the mechanisms underlying the differential transcript abundance in our contrasting AsA lines. As result, various repositories of transcriptome data are now available including NASCArrays, [5] GEO, [6] SMD, [7] ArrayExpress, [8] and AtGenExpress, [9-11], which collectively provide 6,100 microarray data points (7 September 2011). Using this repository, we have searched for the most homologous *PVC* and *NVC* genes in Arabidopsis (*AtPVC* and *AtNVC,* respectively), and we have analyzed using a correlation analysis of the clustering tools in Genevestigator V3 [56] whether different experimental conditions were reported to cause the transcriptional induction or repression of these sets of genes.

The results of our co-expression datasets were analyzed independently for *PVC* and *NVC* genes using this huge data set repository. We were expecting that if the *PVC* and *NVC* set of genes have a collective biological significance they must be regulated by similar conditions in an opposite way. We identified that 25.6% of the *AtPVC* genes were induced by flagellin22 (flag22) (Figure 4A). Importantly, we found that 15.5% of *AtNVC* genes were repressed by flag22 treatment in an independent correlation analysis (Figure 4B). The fact that independent correlation analyses using *AtPVC* and *AtNVC* genes rendered similar treatments in Arabidopsis clearly indicates that these are relevant biological associations.

Early events caused by flag22 treatments include ion fluxes across the plasma membrane and the formation of reactive oxygen species [57,58]. This relationship of *PVC* and *NVC* genes with a ROS-generating agent adds further support to the association of AsA content and the maintenance of the redox state, as deduced from the clustering analysis.

## Conclusion

The differentially expressed genes that correlate with AsA content in tomato fruits identified in the present study suggest a scenario where several genes involved in hormone responses are interlinked to modulate the content of AsA (Figure 6). Cross-talk among different hormones has been reported to regulate several processes in plants: gibberellins and auxin in tomato fruit set [48,49,59]; gibberellins and ABA during seed germination [49,60]; gibberellins and ethylene, in root elongation [49,61]; ABA and ethylene, in the modulation the overall carbon status during early seedling growth and development [62]; brassinosteroids and auxin in the control of lateral root growth [63], etc. Ethylene has been implicated indirectly in the regulation of reduced AsA content in pericarp prior to tomato ripening as the ethylene-insensitive *Nr* mutant accumulates almost 2-fold AsA compared to wild type during Mature green and Breaker stages [26]. ABA has also been connected with the increase of AsA content through promoting its recycling [39]. The co-regulation analyses in Arabidopsis using the *PVC* and *NVC* orthologs identified that these genes are similarly regulated by flag22, whose primary effect is the generation of ROS in response to pathogen attack [57,58]. These results suggest that AsA content in tomato fruit might be critically regulated by hormone interactions, and it is directly related to the oxidative status of the fruit, as previously suggested [64]. However an important aspect that needs to be considered is the likely possibility that part of the AsA in the tomato fruit as has been recently reported. Therefore part of the fruit AsA, in addition of *in situ* biosynthesis, is likely to be provided by source organs complicating even more the identification of AsA determinants [65].

Current approaches have been unfruitful in explaining the natural variability of AsA content in tomato fruit. The combination of different omics approaches, surveying several metabolites, genes, and gene products seems to be the most realistic way of finding out the source(s) of complex traits like AsA content, whose variation among different tomato species is intricately related with hormones [26,39,66]. The exhaustive analyses of the omics data provides a more rational picture of the actual physiological processes determining the AsA fruit content, and would allow to target sets of co-expressed genes to be further studied by functional analyses.

## Methods

### Plant material

A population of 158 Recombinant Inbred Lines (RILs) generated between an inter-specific cross of *Solanum lycopersicum* cv. Moneymaker (MM) and *Solanum pimpinellifollium* (acces. TO-937) were cultivated in a multi tunnel polyethylene greenhouse as well as the parental plants. RILs were generated by repeated selfing of an F2 generation to reach the F_8_ generation by single seed descent procedure. Fruits in red ripe stage were collected during two harvests, in June 2005 and late spring of 2006. Only fruits from trusses 3–4 were collected and during the same time lapse and hour of the day to minimize environmental fluctuations that could affect AsA content. Three independent samples from each RIL and at least three fruits per plant were collected in the harvest 2005 while a pool from all biological replicates was made in harvest 2006. In the first harvest (2005) the fruits were picked at the breaker stage and were kept at room temperature until reaching the light red stage, sliced and immediately frozen in liquid nitrogen and stored at −80°C until analyzed. On the other hand, fruits from the second harvest (2006) were picked at light red stage. Visual inspection was used to cull obvious variants (typically, fruits with peel cracking and showing Yellow Shoulder Disorder as well as parthenocarpic fruit).

### Microarray experiments

Microarray experiments were carried out at Fruit Genomics and Biotechnology lab of the Instituto de Biologia Molecular y Celular de Plantas. Normalization and curation of microarray data was carried out at the Computer Architecture and the Laboratorio de Bioquímica y Biotecnología Vegetal, Universidad de Malaga.

### RNA Extraction and microarray hybridization

Ten out of 158 tomato RILs (*S. lycopersicum* cv. Moneymaker x *S. pimpinellifollium* accession. TO-937) were selected based on their extreme pericarp AsA content (the five with lowest and the five with highest contents) in the harvest 2005 to perform expression analyses. Data were confirmed with harvest 2006. Three replicates per RIL were assayed.

RNA extraction was performed as described previously [67]. RNA amplification and aminoallyl labeling was performed with MessageAmp™ aRNA kit (Ambion) according to manufacturer indications. The AminoAllyl aRNA dye coupling and purification was performed using Megaclear Kit and fragmentation was achieved using RNA Fragmentation Reagents (Ambion) following manufacturer instructions.

For expression analyses a tomato microarray (13,056 spots) was employed (see details at http://www.ibmcp.upv.es).

Each of the RILs RNA as well as two pools, each one comprised of the low- and high-AsA content RILs (Low-pool and High-pool; respectively), was hybridized individually against a control made of the pool of all contrasting RILs (both low- and high-AsA content). The experimental design included a dye-swap, and images were obtained with the *GenePix Pro 6.0*.

### Scanning, data acquisition, filtering, and processing

Expression signal data were read and processed with the standalone software Prep + 07 [68]. All scan acquisitions were performed at normal intensity (PMT GAIN = 730 V × 610 V) with a minimal number of saturated signals (less than 0.55% in all cases). The protocol used in this study includes empty spots that contain a solution of DMSO 50% in order to maintain the rest of values unaltered. Technical replicates were merged into a single data by using the estimated median of their signals, excluding those replicates with a high standard deviation. Data were preprocessed in order to obtain log-ratios following by the filtering of low quality data and the normalizing with *Lowess*. Differential expression was calculated through the mean and standard deviations of the spot distribution of Log_2_(ratio) values, and also defining a global fold change difference and confidence, equivalent to a z-test [68].

The microarray data analyses were performed with TIGR Multiple Experiment Viewer Software v4.6 (http://www.tm4.org) [34] using an expression matrix of 7,351 spots with two or less missing values. KNN impute [69] was used to approximate missing values. Differentially-expressed transcripts were obtained using SAM (*Significant Analysis of Microarray*) [69], implemented as described previously (http://www-stat.stanford.edu/~tibs/SAM/) using a *d* = 1.3 and a fold-change threshold of 1 (as data is represented in a Log_2_ of fold difference, it represents a 2-fold change in expression). A valuable feature of SAM is that it gives estimates of the False Discovery Rate (FDR), which is the proportion of genes likely to have been identified by chance as being significant [70]. Furthermore, SAM is an interactive algorithm that allows the user to eyeball the distribution of the test statistic, and then set thresholds for significance (through the tuning parameter delta) after looking at the distribution [70]. In our experiment, using *d* =1.3 allowed us to select 137 genes with a false rate of 4 genes. Expression profiles were clustered using a Self-Organizing Tree Algorithm [71-73] following Pearson coefficient as metrics and establishing a p-value of 0.05. Blast2GO (http://blast2go.bioinfo.cipf.es/) was used to provide automatic high-throughput annotation, gene ontology (GO) mapping, and categorization of unigenes showing differential expression [35]. Sequences whose annotation was not automatically provided through similarity matching in the NCBI non-redundant NR database, Interpro, or SwissPro databases were annotated according to the Sol Genomic Network database (http://solgenomics.net). In each case, an expectation value threshold of 10^-10^ was used. Functional categorization of genes was performed with the GO slim tool of the Tomato expression database (http://ted.bti.cornell.edu/cgi-bin/TFGD/array/funcat.cgi).

### Statistical analyses

Statistical analyses were performed using Statgraphics Centurion XVI and Microsoft Excel. Interaction between the factors “Year” and “Genotype” was assessed through a two-way ANOVA. Significant differences in the means of ascorbic acid content among the selected RILs and the parental lines were determined using ANOVA and Least Significant Difference (LSD) as *post*-*hoc* test.

### cDNA synthesis and QRT- PCR

Total RNA extracted was used to synthesize single-stranded cDNA using the M-MLV reverse transcriptase (RNase H depleted, Promega GmbH) and oligo(dT) primer according to the manufacturer’s instructions. The PCR amplification was performed with gene-specific primers (Table: List of GOI and the primers used for PCR amplification for cDNA synthesis). GAPDH (glyceraldehyde 3-phosphate dehydrogenase, SGN-U212862) and/or gene SGN-U214197 (C1-QRT), not displaying changes in expression according to microarray analysis, were used to normalize QRT- PCR analyses.

The list of GOI and the primers used for PCR amplification for cDNA synthesis have been: SGN-U220976 (Gene3), Fw: 5’-CTACCTCTGGCCACTCTCAA-3’, Rv: 5’-AACTTCGTCTTTTCCCCATC-3’; SGN-U226214 (Gene6), Fw: 5’-TGCTCTTTTTGCTTCATTTGG-3, ’Rv: 5’- TGGTACAGGCGATAAAATCCTT-3’; SGN-U220900 (Gene7), Fw:5’- TTTTATGGCTATGCCGTCGT-3’, Rv: 5’- GGTCCACAGACGATTCCATT-3’; SGN-U213289 (Gene 12), Fw: 5’- ATGGGGTTGATCATGAATTG-3’, Rv:5’- ATGGGGTTGATCATGAATTG-3’.

The cDNA was amplified using the SYBR-Green PCR Master kit (Applied Biosystems) containing AmpliTaq Gold Polymerase using an iCycler (BioRad) according to the protocol provided by the supplier. QRT- PCR quantification was performed using pBase Plus version 1.5 [74,75].

### AsA determination

AsA content in fruit tissue was determined by using a High-performance liquid chromatography equipment (Jasco) with a reversed-phase column (Gemini® 3 μm C18 Phenomenex, Inc.; Kromasil C18, Scharlau) with ultraviolet detection (254 nm). Tomato pericarp was ground to a fine powder in liquid nitrogen and weighted. AsA extraction was as follows: 1 ml of Extraction buffer (2% m-phosphoric acid, 2 mM EDTA) was added to 0.15 mg of frozen powder. Samples were vortexed till thawing the extraction buffer and were kept on ice for 20 min. They were spun down, filtered and carefully transferred to an HPLC vial to avoid warming out of the sample. The HPLC mobile phase was 0.1 M NaH_2_PO4 and 0.2 mM Na_2_EDTA, pH 3.1 adjusted with ortho-phosphoric acid as previously reported [76].

## Competing interests

The author(s) declare that they have not competing interests.

## Authors’ contributions

VL-S carried out the ascorbic acid determination, expression studies by microarrays and QRT-PCR, data analysis and preparation of the manuscript. AR, data analysis and preparation of the manuscript. VA-S, Arabidopsis data analysis. AM-M, and OT, data analysis of the microarrays. AG supervised the microarray hybridization and data analysis. RF-M, generated the RILs and collected the samples. AB and VV, data analysis and preparation of the manuscript. MAB, project co-ordination and supervision, preparation of the manuscript. All authors read and approved the final manuscript.

## Supplementary Material

Additional file 1 **List of genes differentially expressed between RILs with contrasting content of AsA.** Unigene annotations based on the Sol Genomic Networks web site (http://solgenomics.net), and the accession numbers corresponding to Gene bank best hit and the Arabidopsis orthologs identified using Blast2go [35] are included. Genes are grouped according to the clusters obtained after SOTA analysis. Click here for file

Additional file 2 **Validation of microarray results.** The relative expression of four differentially-expressed genes from the microarray analyses were validated by QRT-PCR. Four genes were selected based on their expression profile between two groups of RILs having the lower (R149 and 29) and the higher (RILs 15 and 64) ascorbic acid contents. *Z*-score are shown in the microarray data. Mean values ± SE are shown. Genes: Gene3, SGN-U220976; Gene6, SGN-U226214; Gene7, SGN-U220900; Gene12, SGN-U213289. GAPDH (acc. U580213) was used to normalize data. Click here for file
