# Supplementary figures and images for: Genetic and genome-wide transcriptomic analyses identify co-regulation of oxidative response and hormone transcript abundance with vitamin C content in tomato fruit
Source: BMC Genomics. 2012 May 14;13:187. doi: 10.1186/1471-2164-13-187 (PMC3462723; doi:10.1186/1471-2164-13-187)

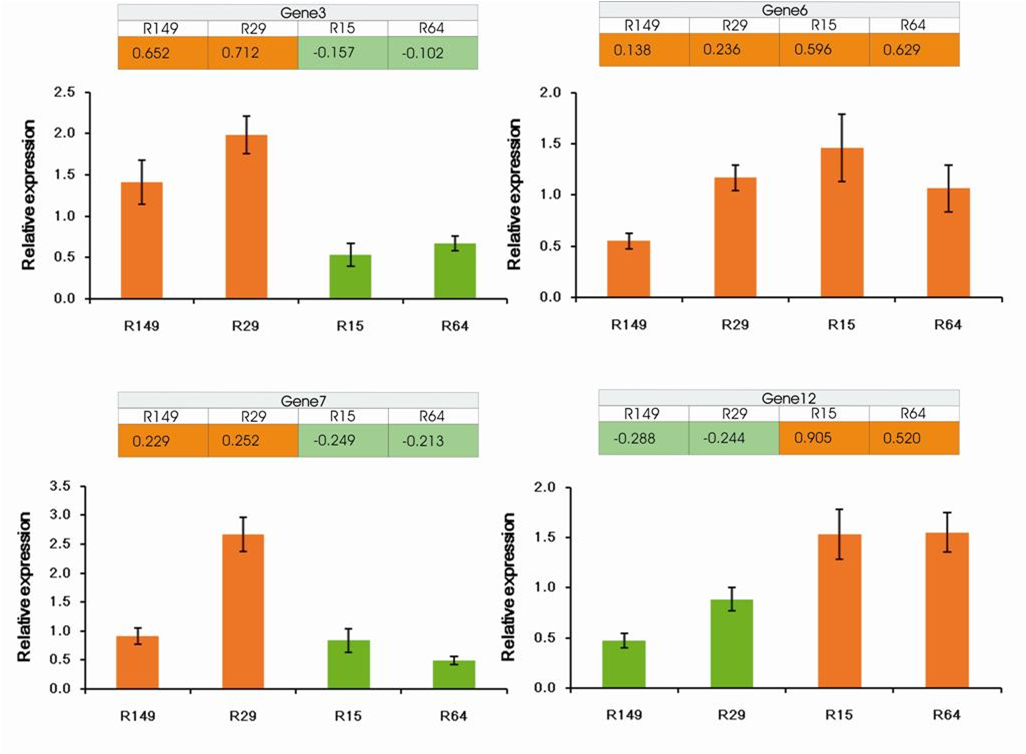

Supplement: Additional file 2 — Validation of microarray results. The relative expression of four differentially-expressed genes from the microarray analyses were validated by QRT-PCR. Four genes were selected based on their expression profile between two groups of RILs having the lower (R149 and 29) and the higher (RILs 15 and 64) ascorbic acid contents. Z-score are shown in the microarray data. Mean values ± SE are shown. Genes: Gene3, SGN-U220976; Gene6, SGN-U226214; Gene7, SGN-U220900; Gene12, SGN-U213289. GAPDH (acc. U580213) was used to normalize data. [file 1471-2164-13-187-S2.jpeg]
